# Use of Causal Inference Methods in Exploring Gerotherapeutic Benefits of FDA-Approved Medications

**DOI:** 10.1093/geroni/igaf122.1577

**Published:** 2025-12-31

**Authors:** Jatupol Kositsawat, George Kuchel, Karen Bandeen-Roche

## Abstract

Randomized controlled trials (RCTs) represent the “gold” standard for validating gerotherapeutics for their potential delaying the onset and progression of varied chronic diseases. However, observational studies designed to guide the design of expensive and lengthy RCTs are subject to nearly unavoidable biases. Causal inference methods permit inference of causal relationships between exposures and outcomes, serving as a methodological link spanning the gap between traditional associational studies and RCTs. Causal inference benefits include ability to move from association to causality, decreased bias, and opportunities for sensitivity analyses. Mendelian randomization (MR), in the development of gerotherapeutics, uses genetic variants strongly associated with a proxy of drug exposure to mimic the drug’s actions. Comparing genotype groups is analogous to comparing different treatment groups in RCTs for the long-term effects on outcomes. This symposium proposal includes a talk by Dr. Kuchel, who will present updated results of our 2022 Aging Cell article “Geroscience-guided repurposing of FDA-approved drugs to target aging: A proposed process and prioritization,” including the first proposal for Cochrane-like assessments of the quality of traditional observational studies, while Dr. Chia-Ling Kuo will focus on Mendelian randomization studies, prioritizing evidence for gerotherapeutic potential based on study design and statistical considerations. Dr. Kositsawat will present MR data evaluating the benefits of metformin in Alzheimer’s disease (AD) prevention. Dr. Karen Bandeen-Roche would be a Discussant to seek common threads between traditional observational epidemiology and causal inference methods. This meeting will highlight this critical topic with a particular emphasis on assessing medications with potential gerotherapeutic benefits. Geroscience Interest Group Sponsored Symposium

